# Supplementary material for: Drug repurposing for aging research using model organisms
Source: Aging Cell. 2017 Jun 16;16(5):1006–15. doi: 10.1111/acel.12626 (PMC5595691; doi:10.1111/acel.12626)
Supplement: Supplementary file 7 — Data S1 Zip‐Archive of all report cards. [file ACEL-16-1006-s007.zip › RC_ALH.pdf]

ALH

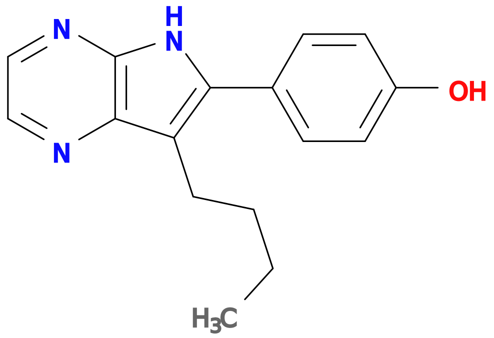

Database identifiers

ChEMBLCompound	CHEMBL75680
DrugBank	DB07364
ZINC	ZINC02540737
eMolecules	2794979

Ranking

	Rank	Score
Drosophila	90/697	0.794
C. elegans	60/591	0.401

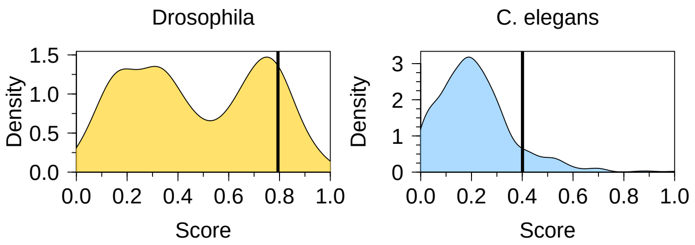

	Ageing implication	Domain conservation	Binding site conservation	Binding affinity	Bioavailability	Lipinski	Promiscuity	Purchasability	Drug approval	**Total**
Drosophila	1.0	0.967	0.918	0.775	(0.9)	0.0	-0.0	0.1	0.075	0.794
C. elegans	1.0	0.965	0.97	0.775	0.312	0.0	-0.0	0.1	0.075	0.401

Names

- 4-(7-butyl-1,5-dihydropyrrolo4,5-bpyrazin-6-ylidene)-1-cyclohexa-2,5-dienone
- 6-PHENYL[5H]PYRROLO[2,3-B]PYRAZINE
- 7-n-Butyl-6-(4-hydroxyphenyl)5Hpyrrolo2,3-bpyrazine
- Aloisine A

Roles

ChEBI entry None has no roles

Status

Approved drug (according to ChEMBL)	No
Classification (according to DrugBank)	experimental
Number of Rule of 5 violations	0
Binding affinity to original target in log units (RF-Score prediction)	6.24
Burns *C. elegans* bioavailability prediction	-0.08

Compound Target Characteristics

Cyclin-dependent-like kinase 5

Best gene implication in ageing for this target family came from gene P48609 annotated in UniProt release 2014_02. Annotation GO 8340 (determination of adult lifespan) was Inferred from Mutant Phenotype

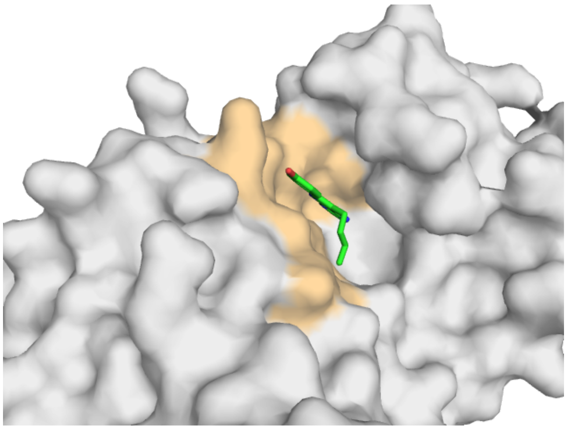

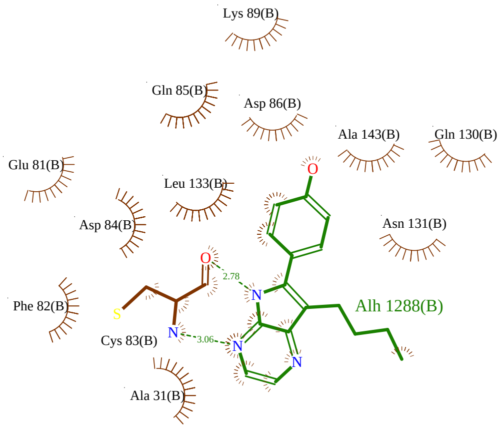

protein	amino acids contacts (binding site)
PDB:1ung:chainA:Q00535	I A E F C D Q D K Q N L A N
PDB:1ung:chainB:Q00535	- A E F C D Q D K Q N L A N
sp:Q00535:CDK5_HUMAN	I A E F C D Q D K Q N L A D
sp:Q03114:CDK5_RAT	I A E F C D Q D K Q N L A D
sp:P49615:CDK5_MOUSE	I A E F C D Q D K Q N L A D
tr:Q543F6:Q543F6_MOUSE	I A E F C D Q D K Q N L A D
sp:P48609:CDK5_DROME	I A E H C D Q D K Q N L A D
tr:G5ECH7:G5ECH7_CAEEL	I A E Y C D Q D K Q N L A D
sp:P17157:PHO85_YEAST	L A E F M D N D K Q N L G D

	whole protein		domain-based		contact-based	
protein	ident	simil	ident	simil	ident	simil
PDB:1ung:chainA:Q00535	1.0	1.0	1.0	1.0	1.0	1.0
PDB:1ung:chainB:Q00535	1.0	1.0	1.0	1.0	0.93	0.86
sp:Q00535:CDK5_HUMAN	1.0	1.0	1.0	1.0	0.93	0.98
sp:Q03114:CDK5_RAT	0.99	1.0	0.99	1.0	0.93	0.98
sp:P49615:CDK5_MOUSE	1.0	1.0	1.0	1.0	0.93	0.98
tr:Q543F6:Q543F6_MOUSE	1.0	1.0	1.0	1.0	0.93	0.98
sp:P48609:CDK5_DROME	0.78	0.93	0.79	0.94	0.86	0.92
tr:G5ECH7:G5ECH7_CAEEL	0.74	0.93	0.75	0.93	0.86	0.97
sp:P17157:PHO85_YEAST	0.54	0.82	0.56	0.83	0.64	0.78

Cdk5 (FBgn0013762) associated phenotypes
neuroanatomy defective, short lived

(Information from FlyBase)

Cdk5 (UniProt:P48609) annotation
Function: Probably involved in the control of the cell cycle. Interacts with D1 and D3-type G1 cyclins. Possible regulator of neuronal differentiation and/or development (By similarity).
Tissue specificity: Abundantly expressed in all adult tissues. Lower levels found in larvae and early embryos. Barely detectable in late embryos.

(Information from UniProt)

cdk-5 (WBGene00000407) associated phenotypes
aldicarb resistant

(Information from WormBase)

cdk-5 (UniProt:G5ECH7) annotation
Function: Proline-directed serine/threonine-protein kinase which, in several motor neurons, promotes the polarized trafficking of synaptic vesicles and dense-core vesicles (DCV). In the ventral nerve cord, phosphorylates lin-10 and thereby prevents lin-10- mediated anterograde trafficking of the glutamate receptor glr-1 (PubMed:17671168, PubMed:21609829). Involved in the inhibition of glr-1 trafficking in hypoxic conditions (PubMed:22252129). In DA motor neurons but not in DB motor neurons, regulates axonal transport of synaptic vesicle precursors by inhibiting dynein-mediated retrograde transport (PubMed:20510931). Regulates the trafficking of dense-core vesicles in DA and DB motor neurons by promoting anterograde trafficking to the axon and preventing dynein-dependent trafficking to the dendrite (PubMed:22699897). May regulate these processes in association with cdka-1/p35 (PubMed:17671168, PubMed:20510931). Activity may be regulated by cyy-1 (PubMed:20510931). Involved in synapse formation during DD motor neuron remodeling by regulating transport of disassembled synaptic material to the new synaptic sites probably by activating the motor protein unc-104/kinesin-3 (PubMed:21609829). Regulates microtubule polarity in the dendrite of DB motor neurons (PubMed:22699897). (PubMed:17671168, PubMed:20510931, PubMed:21609829, PubMed:22252129, PubMed:22699897).
Cofactor: Mg(2+) Evidence=(PubMed:20510931); Note=Binds 2 Mg(2+) ions. (UniProtKB:P24941);
Subunit: Heterodimer composed of a catalytic subunit cdk-5 and a regulatory subunit cdka-1. Interaction with cdka-1 is required for cdk-5 activation. (PubMed:22699897).
Subcellular location: Cytoplasm (PubMed:20510931). Cell projection, dendrite (PubMed:20510931). Note=Localizes predominantly to presynaptic sites and in dendrites as faint puncta. (PubMed:20510931).
Disruption phenotype: Several glr-1-dependent behaviors are affected including an absence of backward locomotion after nose- touching stimuli and a reduction in reverse locomotion (PubMed:17671168). In L4 mutants, incomplete elimination of ventral rab-3-positive synaptic vesicles associated with a delay in the formation of dorsal rab-3-positive synaptic vesicles in DD motor neurons. Normal formation of ventral synapses in DD motor neurons at the L1 stage (PubMed:21609829). In addition, mutants have an increase in anterograde dense-core vesicle trafficking and in the number of plus-end-out microtubules in DB motor neuron dendrites (PubMed:22699897). Reduced sensitivity to the acetylcholine esterase inhibitor aldicarb (PubMed:22699897). (PubMed:17671168, PubMed:21609829, PubMed:22699897).

(Information from UniProt)
